# Regional distribution of carbapenemase-producing *Acinetobacter baumannii* isolates in southern Spain (Andalusia)

**DOI:** 10.1007/s10096-025-05047-2

**Published:** 2025-02-17

**Authors:** Felipe Fernández-Cuenca, Salud Rodríguez-Pallares, Lorena López-Cerero, José Gutiérrez-Fernández, María Fe Bautista, Juan Antonio Sánchez Gómez, Waldo Sánchez-Yebra Romera, Mercedes Delgado, Esther Recacha, Alvaro Pascual

**Affiliations:** 1 University Hospital Virgen Macarena, Seville, Spain; 2 Institute of Biomedicine of Seville, Seville, Spain; 3 University of Seville, Seville, Spain; 4 CIBER de Enfermedades Infecciosas (CIBERINFEC), Instituto de Salud Carlos III, Madrid, Spain; 5 Servicio de Microbiología e Instituto de Investigación Biomédica A Coruña (INIBIC), Complexo Hospitalario Universitario A Coruña, A Coruña, Spain; 6 University Hospital Virgen de Las Nieves, Granada, Spain; 7 La-Inmaculada Hospital, Almería, Spain; 8 UGC Biotechnology, Unit of Microbiology, University Hospital Torrecárdenas, Almería, Spain

**Keywords:** Acinetobacter baumannii, Southern of Spain, KL type, Interregional spread

## Abstract

**Objectives:**

This is the first study conducted in southern Spain to determine i) the population structure (PS) of carbapenem-resistant (CR) *Acinetobacter baumannii* isolates by multilocus sequencing typing (MLST) and core genome MLST (cgMLST) and ii) the association between the sequence type ST and the *bla*_OXA-51_ variant, capsule polysaccharide locus (KL) and lipooligosaccharide outer core locus (OCL) types.

**Methods:**

Of 336 isolates submitted to the Andalusian reference laboratory (PIRASOA; December 2017–2020), 73 were subjected to WGS (MiSeq). The following analyses were performed: bacterial identification (ribosomal MLST), carbapenemase gene detection (Resfinder 4.0), PS delineation (MLST by MLSTfinder 2.0 and cgMLST by Ridom SeqSphere^+^), and KL types and OCL types (Kaptive tool).

**Results:**

The carbapenemases detected were *bla*_OXA-23_ (n = 41), *bla*_OXA-58_ (n = 26), *bla*_OXA-24_ (n = 5), *bla*_OXA-72_ (n = 1) and *bla*_NDM-1_ (n = 2). The PS revealed one major ST2 clone (n = 54) and seven minor ST clones by MLST, and 41 lineages by cgMLST_._ Thirty-five lineages were detected only in a single hospital whereas five lineages were observed in several hospitals and provinces. *bla*_OXA-66_ was the most frequent *bla*_OXA-51_ variant and was mainly associated with the ST2 clone. Eleven KL types and 3 OCL types were assigned, with KL2 (n = 27), KL7 (n = 16) and OCL1 being the most frequent.

**Conclusions:**

The PS of CR *A. baumannii* in Andalusia is characterized by a dominant ST2/*bla*_OXA-23_ clone and several lineages, showing local spread of lineages in most hospitals, and intercenter or interregional spread of a few lineages. Single-locus *bla*_OXA-51-like_ typing and KL typing may be useful as complementary preliminary typing tool.

**Supplementary Information:**

The online version contains supplementary material available at 10.1007/s10096-025-05047-2.

## Introduction

Carbapenem-resistant *A. baumannii* (CR-Ab) is one of the most important nosocomial pathogens worldwide [1]. The main carbapenem resistance mechanism described in CR-Ab is the acquisition of OXA-type carbapenemase genes [2]. The most frequently reported carbapenemase genes worldwide are *bla*_OXA-23_, *bla*_OXA-24/40_ and *bla*_OXA-58_ [3, 4]. It has been observed that the naturally occurring *bla*_OXA-51_ of *A. baumannii* is a useful target for preliminary identification of some *Acinetobacter* spp. and for differentiation of some international clones (IC) of CR-Ab [4, 5].

From an epidemiological point of view, CR-Ab isolates represent a serious global health problem due to their ability to cause outbreaks [1–3]. This applies particularly to isolates belonging to certain IC lineages, such as IC-2 [5]. The introduction of typing methods based on large-scale whole genome sequencing (WGS) has been a major advance in molecular epidemiological research [6]. WGS is currently regarded as the gold standard for bacterial genotyping, offering greater discriminatory power than pulsed-field gel electrophoresis (PFGE), previously considered as the gold standard, and multilocus sequencing typing (MLST), which are widely used for delineating the population structures of bacteria [6–8]. Core genome (cgMLST) and whole genome (wgMLST) schemes, as well as genotyping methods based on sequencing the capsular polysaccharide locus (KL) and the oligosaccharide locus (OCL) have been developed for *A. baumannii* [7–11]. While these genotyping techniques have the potential to be useful for characterizing the population structure of CP-Ab at a regional level, the available experience is still limited and results inconclusive.

The primary objective of this study was to describe by MLST and cgMLST the population structure and clonal distribution of clinical and environmental CR-Ab isolates detected in Andalusia over a 4-year period. The association between the ST and the *bla*_OXA-51-like_ variant, KL type and OCL type was also investigated.

## Material and methods

### Bacterial isolates and identification

Three hundred and thirty-six CR-Ab isolates, collected from surveillance (51%), clinical (48%) and environmental samples (1%) of patients attended in 16 hospitals in Andalusia (Spain) between December 2017 and 2020 were submitted to the Andalusian reference laboratory for antimicrobial reference laboratory for antimicrobial resistance and molecular epidemiology typing (PIRASOA). Submission of isolates was voluntary and not all hospitals submitted all isolates that were detected during this period. The mean annual incidence density of CR-Ab during this period was < 0.01 for 14 out of 16 hospitals (Table S1). In the remaining two hospitals, it was 0.64 for HHO, the Hospital La Inmaculada, in Almeria) and 0.24 for HIE (Hospital Infanta Elena, in Huelva). The preliminary identification of isolates was performed by matrix-assisted laser desorption/ionization time-of-flight mass spectrometry, (MALDI-TOF MS) (MALDI Biotyper CA system; Bruker Daltonics, Madrid, Spain) and detection of *bla*_OXA-51_ by PCR amplification [12].

### Carbapenem susceptibility testing and detection of carbapenemase production

Susceptibility to three carbapenems (ertapenem, imipenem and meropenem) was performed using MicroScan NMDRM1 panels (Beckman Coulter, Inc, Madrid, Spain). Susceptibility testing for imipenem and meropenem was also performed using gradient strips (Etest®, Biomérieux). For phenotypic detection of carbapenemase production, the combined disc test was performed on imipenem with carbapenemase inhibitors (Rosco, Madrid, Spain) and the β CARBA test (BioRad, Madrid, Spain). Initial detection of carbapenemase-encoding genes was performed by end-point PCR, using primers specific for *bla*_OXA-23-like_, *bla*_OXA-24/40-like_ and *bla*_OXA-58-like_ [12]_._

### Whole genome DNA sequencing (WGS)

The Nextera XT DNA Library Preparation Kit (Illumina GmbH, Germany) and the MiSeq Reagent Kit V3 (600 cycles) were used to prepare DNA libraries for sequencing on the Illumina MiSeq sequencer (2 × 300 paired end reads). Reads were quality filtered and de novo assembled using the CLC Genomics Workbench v10 (Qiagen). Table S2 shows the results of the QC analysis and the percentage of cgMLST good targets of *Acinetobacter* Ridom scheme. Annotation of resistance determinants in the assemblies, including *bla*_OXA-51-like_ detection and characterization, was performed using the ResFinder tool, version 4.0 (https://cge.food.dtu.dk/services/ResFinder/) and the Comprehensive Antibiotic Resistance Database (CARD; https://card.mcmaster.ca/) were used. Final bacterial identification was performed using the ribosomal MLST database (https://pubmlst.org/species-id) and *bla*_OXA-51-like_ gene typing [4].

### Molecular typing

For all isolates, the initial clonal relationship was determined by PFGE with ApaI [13]. PFGE patterns were analyzed using Bionumerics version 8.1.1 software and the Dice coefficient. Isolates differing by 1 band or more were selected for NGS (Fig. S1). Assemblies were used for further MLST typing using MLSTfinder version 2.0 (https://cge.food.dtu.dk/services/MLST/). The core genome sequence type (cgST) was determined using Ridom SeqSphere^+^ software based on the scheme of 2390 alleles for the core genome and 1083 alleles for the accessory genome. *A. baumannii* ACICU was included as the reference genome to characterize the allelic profile of isolates on a gene-by-gene basis. To investigate the relatedness of isolates, a minimum spanning tree was constructed based on differences in cgMLST allelic profiles, and including missing values in the UPGMA algorithm. According to RIdom SeqSphere scheme, isolates showing ≤ 9 allelic differences were considered highly related. KL and OCL typing were performed using the Kaptive tool (https://kaptive-web.erc.monash.edu/). Phylogenetic trees were visualized using the Interactive Tree Of Life (iTOL) (http://itol.embl.de). Additionally, Andalusian genomes were compared with those of a recent Portuguese collection of *A. baumannii* isolates detected between 2005–2019 [14].

## Results

### Bacterial identification and detection of carbapenemase production

Of the 336 isolates tested, 335 were identified as *A. baumannii* and one was identified as *Acinetobacter pittii* by Maldi TOF and rMLST. All *A. baumannii* isolates were PCR-positive for *bla*_OXA-51-like_ whereas the *A. pittii* isolate was PCR-negative for *bla*_OXA-51-like._

**Fig. 1:**
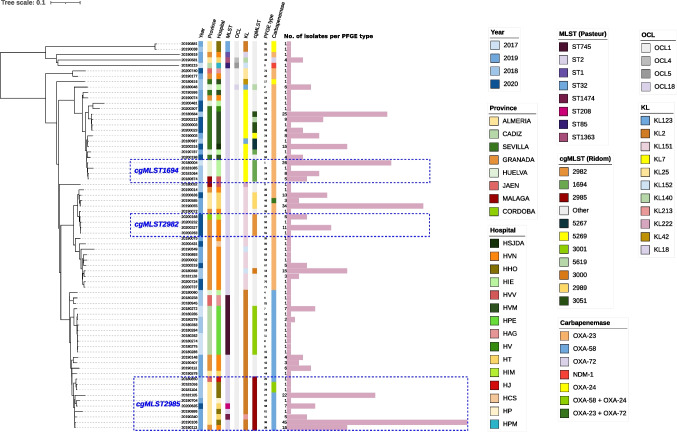
Dendrogram generated from the scheme that combines 1083 accessory genes and 2390 cgMLST genes (Ridom SeqSphere +). A box with dotted lines indicates isolates with the same cgMLST detected in different hospitals in different provinces

The most frequently detected carbapenemase gene was *bla*_OXA-23-like_ (60%; n = 203 isolates, found in 9 hospitals), followed by *bla*_OXA-58-like_ (37%; n = 124 isolates, found in 8 hospitals), and *bla*_OXA-24/40-like_ (5%; n = 18 isolates, found in 2 hospitals). Two isolates (0.6%) were only PCR-positive for *bla*_NDM-like_. Co-detection of different carbapenemase genes was observed only in isolates carrying *bla*_OXA-24/40-like_ and *bla*_OXA-58-like_ (n = 10, 3%) or *bla*_OXA-23-like_ (n = 1, 0.3%). As shown in Fig. 1, the majority of the 336 isolates were collected in 4 provinces and 6 hospitals: 82% of *bla*_OXA-23-like_ isolates in HVN, HVM, HIE and HT, 88% of *bla*_OXA-58-like_ in HHO, HVN, HPE and HT, and 94% of *bla*_OXA-24/40-like_ isolates in HHO and HT.

A subset of 73 isolates (72 *A. baumannii* and one *A. pittii*) representing 73 *ApaI*-PFGE types were selected and subjected to WGS with further identification of carbapenemase-encoding allelic variants, molecular genotyping by MLST and cgMLST, *bla*_OXA-51-like_ typing, KL typing and OCL typing.

The carbapenemase allelic variants were distributed as follows: *bla*_OXA-23_ (n = 41, 56%), *bla*_XA-58_ (n = 26, 36%), *bla*_OXA-24_ (n = 5, 7%), *bla*_NDM-1_ (n = 2, 3%) and *bla*_OXA-72_ (n = 1, 1%) and. Co-detection of two carbapenemase genes was observed in only two *bla*_OXA-58_ and *bla*_OXA-24-_ carrying isolate and one *bla*_OXA-23_ and *bla*_OXA-72-_ carrying isolate.

*A. baumannii* isolates were assigned by MLST to one of the following eight sequence types or clones: ST2, ST745, ST32, ST1474, ST1363, ST1, ST208 or ST85 (Fig. 1). Two sequence types accounted for 90% of the 73 sequenced isolates: ST2 (n = 54, 75%) and ST745 (n = 11, 15%). Isolates belonging to the ST2 clone were detected in 13 (66%) of 16 hospitals, and most of these isolates harbored *bla*_OXA-23_ (n = 40, 74%) or *bla*_OXA-58_ (n = 13, 24%). In contrast, isolates of the ST745 clone were found in only two hospitals, and carried *bla*_OXA-58._

**Fig. 2:**
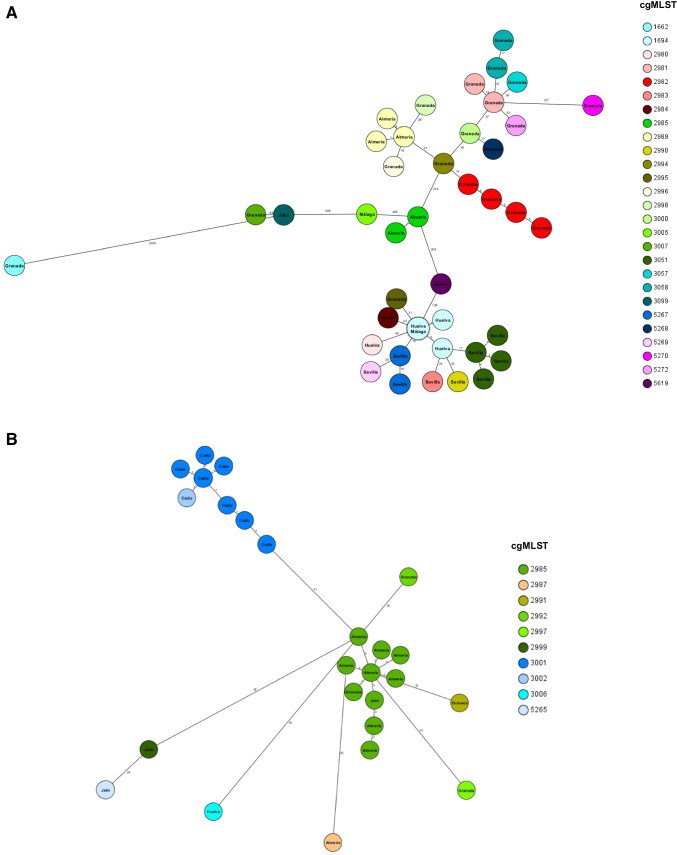
Minimum spanning tree for *bla*_OXA-23_ isolates (panel A) and *bla*_OXA-58_ (panel B) isolates based on cgMLST using the `pairwise ignore missing values´ during distance calculations. The numbers on the connecting lines illustrate the numbers of target genes with different alleles. Different colors of isolates indicate the assigned cgMLST

A percentage of 95.0–99.6% of good targets were obtained using cgMLST Ridom scheme. As shown in Fig. 1 and 2, the population structure determined by cgMLST revealed 41 different cgMLSTs or lineages. Twenty-six lineages were identified in 100% of *bla*_OXA-23-_bearing isolates, whereas only 9 lineages were observed in 46% of *bla*_OXA-58-_carrying isolates. The 26 lineages assigned to *bla*_OXA-23-_bearing isolates were identified in 10 hospitals and 6 provinces (Fig. 1 and 2). During the 3-year period, 21 lineages were observed only once, while 5 lineages were observed in more than one hospital and/or province. In terms of the number of allele differences in the core genome of the lineages, fewer than 10 allele differences were observed in 2 lineages in hospitals in two different provinces each. The same lineage was detected in two hospitals in the city of Seville (10 allele differences in the core genome) and another lineage in two hospitals in the city of Granada (13 allele differences in the core genome). The 9 lineages assigned to *bla*_OXA-58-_bearing isolates were identified in 8 hospitals and 5 provinces (Fig. 1 and 2). Eight lineages were observed only once, and one lineage was observed in five hospitals. Less than 10 alleles of difference in the core genome between lineages were detected in isolates from 4 hospitals in 3 different provinces. Six strains belonged to the ST2 clone and the other 2 to single locus variants of the ST2 clone (ST208 and ST1474). There was a poor correlation between cgMLST and PFGE, especially for cgMLST 2985, 3001, and 1694 which were assigned to 10, 8 and 5 different pulsotypes, respectively. When comparing the Andalusian genomes with the collection of Portuguese isolates, none of the latter shared a cgMLST with those from our region (Fig. S2).

Seven different allelic variants of *bla*_OXA-51-like_ were identified among *A. baumannii* isolates (*bla*_OXA-66,_ *bla*_OXA-67,_ *bla*_OXA-69,_ *bla*_OXA-94,_ *bla*_OXA-100,_ *bla*_OXA-109,_ and *bla*_OXA-508_). Of these variants, *bla*_OXA-66_ was by far the most frequently detected (n = 62; 87%), and was carried by all ST2 isolates harboring *bla*_OXA-23_ or *bla*_OXA-58_, as well as by ST208 and ST745 isolates harboring *bla*_OXA-58_. The remaining five minor *bla*_OXA-51-like_ variants were associated with unique STs (Fig. 1). *bla*_OXA-421_ was detected in the *A. pittii* isolate*.*

A total of eleven KL types were observed (Fig. 1). A low level of discrimination in the KL typing was observed due to the presence of 6 KL types that contained more than 1 cgMLST. The majority (83%) of the KL types observed were classified as either KL2, KL151 or KL7. The KL2 type was the most frequent (n = 27, 38%) and ubiquitous, being observed in 96% of isolates harboring *bla*_OXA-58._ The KL2 type was observed in isolates from 4 provinces and belonged to 4 STs and 11 cgMLSTs. All 11 isolates assigned to ST745 were of the KL2 type (Fig. 1). All 17 isolates with the KL151 type were found in two eastern provinces, carried *bla*_OXA-23_ (41% of *bla*_OXA-23_ isolates)_,_ belonged to the ST2 clone, and were represented by 11 different cgMLSTs. The 16 isolates with the KL7 type were from two western provinces of Andalusia and also carried *bla*_OXA-23_ (39% of *bla*_OXA-23_ isolates). They were assigned to the ST2 clone and were represented by 10 cgMLSTs.

Four OCL types were observed, the most frequent being OCL1, which was identified in 69 (96%) isolates.

## Discussion

This study presents the findings of a recent population structure analysis of CR-Ab performed in the region of Andalusia (surface area 87,600 Km^2^), southern Spain. In our study, the population structure of CR-Ab isolates defined by MLST and using the Pasteur scheme revealed the presence of a dominant high-risk clone, composed mainly of *bla*_OXA-23_ isolates belonging to ST2, included in the clonal complex 2 (CC2), which were disseminated in most Andalusian hospitals and provinces. These results agree with previous studies performed in Spain, other countries of Europe, Asia and the USA [3, 15, 16]**,** revealing the significant potential of ST2/*bla*_OXA-23_ isolates for global dissemination in the nosocomial environment [2, 3, 16, 17]. Also worth highlighting is the presence of additional minor clones within CC2, especially ST32 and ST1474, which are single-locus variants of the *rpoB* allele.

To the best of our knowledge, this is the first study to evaluate the ability of cgMLST to define and characterize the population structure of CR-Ab in all the hospitals in our region, as well as its practical value in investigating the regional and interregional spread of CR-Ab in Andalusia. The application of this genotyping method showed that most CR-Ab isolates at each hospital were of local origin, but that this was in parallel with the spread of a single lineage between hospitals. On the other hand, we did not find any genetic relationship between Portuguese and Andalusian isolates, despite the geographical proximity. The epidemiological situation of CR-Ab in Andalusia is consistent with the findings of the international multicenter study performed by Lötsch et al. in nine European countries, which reported regional or inter-regional spread as well as endemicity in seven countries [18]. However, in our study there were some discrepancies between MLST sequence types and the Ridom cgMLST scheme. In the case of the 8 genomes of the cgMLST2985 lineage, six genomes belonged to sequence type ST2 and the other 2 genomes belonged to two single locus variants of ST2. To avoid these discrepancies, the algorithm for new cgMLST assignment could consider the changes in the MLST scheme loci. On the other hand, there was a poor correlation between cgMLST and PFGE pulsotypes, suggesting that the cgMLST scheme used has a lower discriminatory power than that of PFGE.

Our results corroborate those of previous studies, which indicate that *bla*_OXA-23_ and, to a lesser extent, *bla*_OXA-58_ were widely distributed in most Andalusian hospitals and provinces [2, 3, 5, 16]. Part of its capacity for dissemination may be attributed in part to the association of *bla*_OXA-23_ with various mobile genetic platforms containing IS*Aba*-type insertion sequences and/or transposons, as described previously [3, 19]. It is noteworthy that, in the four hospitals that accounted for more than 70% of isolates, isolates carrying different *bla*_OXA_ were found simultaneously_._ This event is not common in CR-Ab. In one of these 4 hospitals, in the same year, *bla*_OXA-58_, *bla*_OXA-23_ and *bla*_OXA-72_ genes were detected and could explain the co-detection of both *bla*_OXA-23_ with *bla*_OXA-72_ and *bla*_OXA-58_ with *bla*_OXA-24_ in some isolates_,_ probably due to the mobilization by horizontal transfer of plasmids carrying genes encoding OXA-type carbapenemases [20].

In line with previous studies, the intrinsic *bla*_OXA-66_ allelic variant was associated with the dominant ST2 clone, whereas the minor variants *bla*_OXA-100_, *bla*_OXA-69_ and *bla*_OXA-94_ were associated with the ST32, ST1 and ST85 clones, respectively [5]. This finding is in agreement with previous studies, which have suggested that single-locus *bla*_OXA-51-like_ sequence-based typing is a useful preliminary typing method, particularly for local epidemiological investigations, as it is able to discriminate between unrelated ST clones [4]. However, it is recommended that *bla*_OXA-51-like_ sequencing be employed in conjunction with other typing methods, as some plasmid-encoded *bla*_OXA-51-like_ gene variants may be carried by certain *Acinetobacter* non-*baumannii* species [21].

Capsular polysaccharides and lipopolysaccharides have been associated with carbapenem resistance, virulence, disease severity, and mortality in *A. baumannii* [22]. However, there is very little information on the usefulness of capsular typing in CR-Ab, particularly in the context of sequence-based K and O locus typing. In our study, capsular type KL2 was the most frequent KL type, which is in line with previous studies where KL2 was the dominant KL type [23], which suggests that this capsule may confer some kind of advantage to CR-Ab compared to other less frequent capsular types, as has been previously reported [23, 24]. Furthermore, KL typing showed much greater discriminatory power than OCL typing, which is also in agreement with previous studies, indicating that KL typing could be a valuable tool for CR-Ab typing [11, 22, 23]. However, our results show that KL typing correlates weakly with ST when using the Pasteur scheme or the cgST scheme of Higgins et al. are employed. The limited correlation between KL type and ST using the Pasteur scheme differs from the results of Luo YC et al. obtained using the Oxford scheme on isolates from Taiwan, where the major KL types were associated with ST and suggesting that KL typing could be a useful tool for studying the population structure of *A. baumannii*, especially when used in conjunction with other genotyping assays [25]. The lack of association between KL type and ST could be explained by the greater discriminatory power of the Oxford scheme compared to the Pasteur scheme [25, 26]. Therefore, we are unable to exclude the possibility that KL type is associated with the ST clone of our isolates according to the Oxford scheme. The main value of KL typing may be to distinguish ST2/*bla*_OXA_-_23_ isolates from ST2/*bla*_OXA_-_58,_ as well as from isolates with an ST other than ST2.

The main strength of our study is its use of a well-characterized collection of CR-Ab isolates from Andalusia (87,600Km^2^), one of the largest regions of Spain. However, the present study has some limitations. The most important is that it was not mandatory to send samples to our reference laboratory. Therefore, it is possible that the real prevalence of isolates, *bla*_OXA-type_ carbapenemase genes, ST clones and cgST lineages may differ from those observed in the present study. However, the majority of isolates came from the two hospitals with the highest prevalence. Another important limitation is the lack of epidemiological data, which would be very useful in interpreting the results and conclusions of this study.

In conclusion, the results of our study show that ST2 is the dominant clone and *bla*_OXA-23_ the most prevalent carbapenemase in Andalusia. The population structure of *A. baumannii* determined by cgMLST shows that most CR-Ab isolates were disseminated locally, whereas a few lineages were involved in interhospital and/or interregional spread. While single-locus *bla*_OXA-51-like_ typing and capsular KL typing are much less discriminant than ST and cgMLST, they may serve as useful complementary typing tools in the preliminary characterization of the population structure of *A. baumannii*.

## Supplementary Information

Below is the link to the electronic supplementary material.

Fig. 3 Supplementary file1 Figure S1. Distribution of the ApaI-PFGE types among 336 Acinetobacter spp. isolates selected for this study.

High resolution image (TIFF 28318 KB)

Supplementary file2 Figure S2. Dendrogram generated with A. baumannii isolates from Andalucía and Portugal using the cgMLST scheme of Ridom SeqSphere+. (PDF 35 KB)

Supplementary file3 Table S1. Incidence density of carbapenem-resistant A. baumannii isolates in Andalusia (Spain) and number of submitted isolates to the Regional Reference laboratory PIRASOA during December 2017-2020. Legend of Table S1 *No. of inpatients with A. baumannii infection/colonisation x 1000/No. of total stays during the year, according to PIRASOA program reported data (htpp://pirasoa.iavante.es/course/view.php?id=3&section=2). NR= not reported to the PIRASOA program; SD= standard deviation. (DOCX 25.2 KB)

Supplementary file4 Table S2. Assembly summary report for the 73 genomes of Acinetobacter spp., including 72 isolates of A. baumannii and one isolate of A. pittii. Legend of Table S2 *Isolate of A. pittii. (DOCX 25.0 KB)

## Data Availability

No datasets were generated or analysed during the current study.

## References

[1] Miller WR, Arias CA (2024) ESKAPE pathogens: antimicrobial resistance, epidemiology, clinical impact and therapeutics. Nat Rev Microbiol 22(10):598–616 38831030 10.1038/s41579-024-01054-w PMC13147291

[2] Müller C, Reuter S, Wille J, Xanthopoulou K, Stefanik D, Grundmann H, Higgins PG, Seifert H (2023) A global view on carbapenem-resistant *Acinetobacter baumannii*. mBio 14(6):e0226023 37882512 10.1128/mbio.02260-23 PMC10746149

[3] Castanheira M, Mendes RE, Gales AC (2023) Global Epidemiology and Mechanisms of Resistance of *Acinetobacter baumannii-calcoaceticus* Complex. Clin Infect Dis 76(Suppl 2):S166–S178 37125466 10.1093/cid/ciad109 PMC10150277

[4] Pournaras S, Gogou V, Giannouli M, Dimitroulia E, Dafopoulou K, Tsakris A, Zarrilli R (2014) Single-locus-sequence-based typing of blaOXA-51-like genes for rapid assignment of *Acinetobacter baumannii* clinical isolates to international clonal lineages. J Clin Microbiol 52(5):1653–1657 24622099 10.1128/JCM.03565-13 PMC3993655

[5] Li J, Li Y, Cao X, Zheng J, Zhang Y, Xie H, Li C, Liu C, Shen H (2023) Genome-wide identification and oxacillinase OXA distribution characteristics of Acinetobacter spp. based on a global database. Front Microbiol 1(14):1174200 10.3389/fmicb.2023.1174200 PMC10267304 37323896

[6] Simar SR, Hanson BM, Arias CA (2021) Techniques in bacterial strain typing: past, present, and future. Curr Opin Infect Dis 34(4):339–345 34039880 10.1097/QCO.0000000000000743 PMC9245535

[7] Forde BM, Bergh H, Cuddihy T, Hajkowicz K, Hurst T, Playford EG, Henderson BC, Runnegar N, Clark J, Jennison AV, Moss S, Hume A, Leroux H, Beatson SA, Paterson DL, Harris PNA (2023) Clinical Implementation of Routine Whole-genome Sequencing for Hospital Infection Control of Multi-drug Resistant Pathogens. Clin Infect Dis 76(3):e1277–e1284 36056896 10.1093/cid/ciac726

[8] Dekker JP, Frank KM (2016) Next-Generation Epidemiology: Using Real-Time Core Genome Multilocus Sequence Typing To Support Infection Control Policy. J Clin Microbiol 54(12):2850–2853 27629902 10.1128/JCM.01714-16 PMC5121370

[9] Higgins PG, Prior K, Harmsen D, Seifert H (2017) Development and evaluation of a core genome multilocus typing scheme for whole-genome sequence-based typing of *Acinetobacter baumannii*. PLoS ONE 12(6):e0179228 28594944 10.1371/journal.pone.0179228 PMC5464626

[10] Venditti C, Vulcano A, D’Arezzo S, Gruber CEM, Selleri M, Antonini M, Lanini S, Marani A, Puro V, Nisii C, Di Caro A (2019) Epidemiological investigation of an *Acinetobacter baumannii* outbreak using core genome multilocus sequence typing. J Glob Antimicrob Resist 17:245–249 30553929 10.1016/j.jgar.2018.11.027

[11] Wyres KL, Cahill SM, Holt KE, Hall RM, Kenyon JJ (2020) Identification of *Acinetobacter baumannii* loci for capsular polysaccharide (KL) and lipooligosaccharide outer core (OCL) synthesis in genome assemblies using curated reference databases compatible with Kaptive. Microb Genom 6(3):e000339 32118530 10.1099/mgen.0.000339 PMC7200062

[12] Woodford N, Ellington MJ, Coelho JM, Turton JF, Ward ME, Brown S, Amyes SG, Livermore DM (2006) Multiplex PCR for genes encoding prevalent OXA carbapenemases in *Acinetobacter* spp. Int J Antimicrob Agents 27(4):351–353 16564159 10.1016/j.ijantimicag.2006.01.004

[13] Seifert H, Dolzani L, Bressan R, van der Reijden T, van Strijen B, Stefanik D, Heersma H, Dijkshoorn L (2005) Standardization and interlaboratory reproducibility assessment of pulsed-field gel electrophoresis-generated fingerprints of *Acinetobacter baumannii*. J Clin Microbiol 43(9):4328–4335 16145073 10.1128/JCM.43.9.4328-4335.2005 PMC1234071

[14] Domingues R, Oliveira R, Silva S, Araújo D, Almeida C, Cho GS, Franz CMAP, Saavedra MJ, Azeredo J, Oliveira H (2024) Molecular Detection of Carbapenemases in *Acinetobacter baumannii* Strains of Portugal and Association With Sequence Types, Capsular Types, and Virulence. Clin Ther 46(12):e9–e15 39384436 10.1016/j.clinthera.2024.09.005

[15] Villalón P, Valdezate S, Medina-Pascual MJ, Rubio V, Vindel A, Saez-Nieto JA (2011) Clonal diversity of nosocomial epidemic *Acinetobacter baumannii* strains isolated in Spain. J Clin Microbiol 49(3):875–882 21177889 10.1128/JCM.01026-10 PMC3067678

[16] Lasarte-Monterrubio C, Guijarro-Sánchez P, Alonso-Garcia I, Outeda M, Maceiras R, González-Pinto L, Martínez-Guitián M, Fernández-Lozano C, Vázquez-Ucha JC, Bou G, Arca-Suárez J, Beceiro A (2024) Epidemiology, resistance genomics and susceptibility of *Acinetobacter* species: results from the 2020 Spanish nationwide surveillance study. Euro Surveill 29(15):2300352 38606569 10.2807/1560-7917.ES.2024.29.15.2300352 PMC11010588

[17] Diancourt L, Passet V, Nemec A, Dijkshoorn L, Brisse S (2010) The population structure of *Acinetobacter baumannii*: expanding multiresistant clones from an ancestral susceptible genetic pool. PLoS ONE 5(4):e10034 20383326 10.1371/journal.pone.0010034 PMC2850921

[18] Lötsch F, Albiger B, Monnet DL, Struelens MJ, Seifert H, Kohlenberg A, European Antimicrobial Resistance Genes Surveillance Network (EURGen-Net) carbapenem-resistant Acinetobacter baumannii capacity survey group; EURGen-Net carbapenem-resistant Acinetobacter baumannii capacity survey group (2020) Epidemiological situation, laboratory capacity and preparedness for carbapenem-resistant *Acinetobacter baumannii* in Europe, 2019. Euro Surveill 25(45):2001735 33183407 10.2807/1560-7917.ES.2020.25.45.2001735 PMC7667627

[19] Poirel L, Nordmann P (2006) Genetic structures at the origin of acquisition and expression of the carbapenem-hydrolyzing oxacillinase gene blaOXA-58 in *Acinetobacter baumannii*. Antimicrob Agents Chemother 50(4):1442–1448 16569863 10.1128/AAC.50.4.1442-1448.2006 PMC1426978

[20] Salgado-Camargo AD, Castro-Jaimes S, Gutierrez-Rios RM, Lozano LF, Altamirano-Pacheco L, Silva-Sanchez J, Pérez-Oseguera Á, Volkow P, Castillo-Ramírez S, Cevallos MA (2020) Structure and Evolution of *Acinetobacter baumannii* Plasmids. Front Microbiol 11:1283 32625185 10.3389/fmicb.2020.01283 PMC7315799

[21] Lee YT, Kuo SC, Chiang MC, Yang SP, Chen CP, Chen TL, Fung CP (2012) Emergence of carbapenem-resistant non-baumannii species of Acinetobacter harboring a blaOXA-51-like gene that is intrinsic to A. baumannii. Antimicrob Agents Chemother 56(2):1124–1127 22083478 10.1128/AAC.00622-11 PMC3264228

[22] Hsieh YC, Wang SH, Chen YY, Lin TL, Shie SS, Huang CT, Lee CH, Chen YC, Quyen TLT, Pan YJ (2020) Association of capsular types with carbapenem resistance, disease severity, and mortality in *Acinetobacter baumannii*. Emerg Microbes Infect 9(1):2094–2104 32912064 10.1080/22221751.2020.1822757 PMC7534287

[23] Kostyanev T, Xavier BB, García-Castillo M, Lammens C, Bravo-Ferrer Acosta J, Rodríguez-Baño J, Cantón R, Glupczynski Y, Goossens H, EURECA/WP1B Group (2021) Phenotypic and molecular characterizations of carbapenem-resistant *Acinetobacter baumannii* isolates collected within the EURECA study. Int J Antimicrob Agents 57(6):106345 33887390 10.1016/j.ijantimicag.2021.106345

[24] Oliveira H, Mendes A, Fraga AG, Ferreira A, Pimenta AI, Mil-Homens D, Fialho AM, Pedrosa J, Azeredo J (2019) K2 Capsule Depolymerase Is Highly Stable, Is Refractory to Resistance, and Protects Larvae and Mice from *Acinetobacter baumannii* Sepsis. Appl Environ Microbiol 85(17):e00934-e1019 31227554 10.1128/AEM.00934-19 PMC6696962

[25] Luo YC, Hsieh YC, Wu JW, Quyen TLT, Chen YY, Liao WC, Li SW, Wang SH, Pan YJ (2022) Exploring the association between capsular types, sequence types, and carbapenemase genes in *Acinetobacter baumannii*. Int J Antimicrob Agents 59(1):106470 34757135 10.1016/j.ijantimicag.2021.106470

[26] Gaiarsa S, BatistiBiffignandi G, Esposito EP, Castelli M, Jolley KA, Brisse S, Sassera D, Zarrilli R (2019) Comparative Analysis of the Two *Acinetobacter baumannii* Multilocus Sequence Typing (MLST) Schemes. Front Microbiol 10:930 31130931 10.3389/fmicb.2019.00930 PMC6510311

