# Supplementary figures and images for: Regional distribution of carbapenemase-producing Acinetobacter baumannii isolates in southern Spain (Andalusia)
Source: Eur J Clin Microbiol Infect Dis. 2025 Feb 17;44(5):1069–76. doi: 10.1007/s10096-025-05047-2 (PMC12062160; doi:10.1007/s10096-025-05047-2)

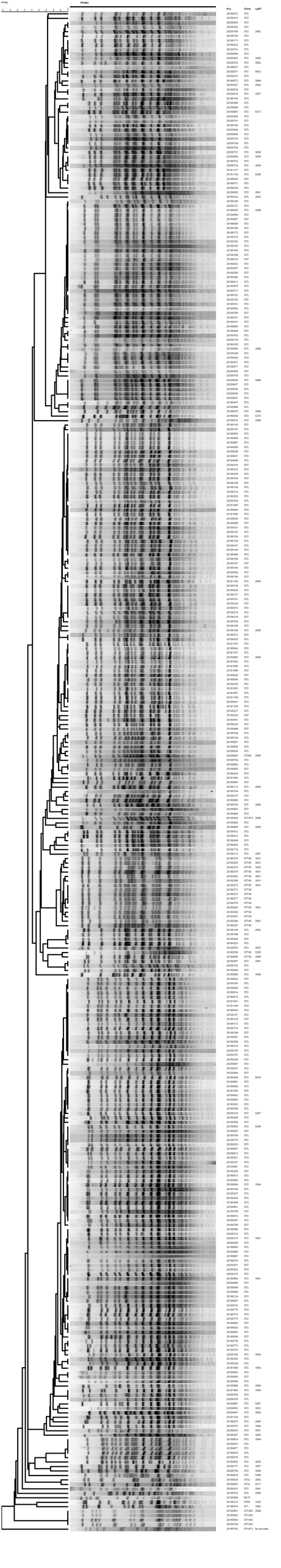

Supplement: Supplementary file 1 — Supplementary file1 Figure S1. Distribution of the ApaI-PFGE types among 336 Acinetobacter spp. isolates selected for this study. [file 10096_2025_5047_Fig3_ESM.png]

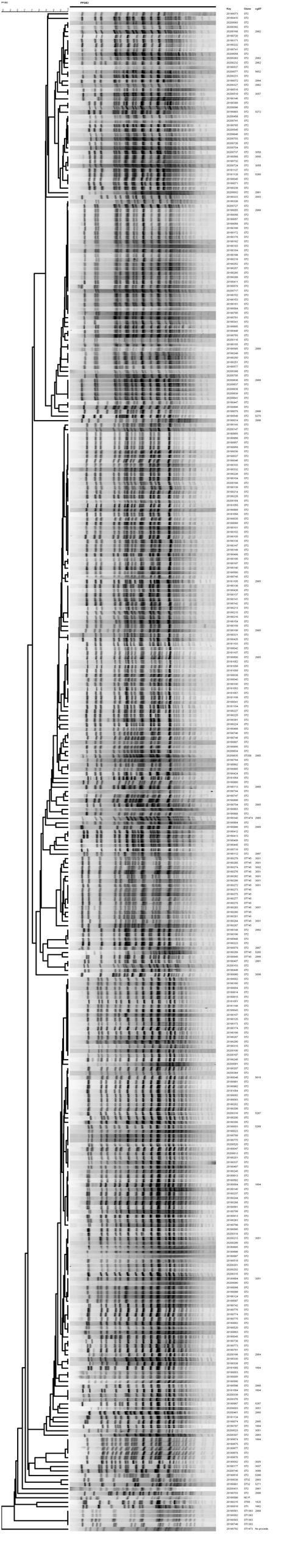

Supplement: Supplementary file 2 — High resolution image (TIFF 28318 KB) [file 10096_2025_5047_MOESM1_ESM.tif]

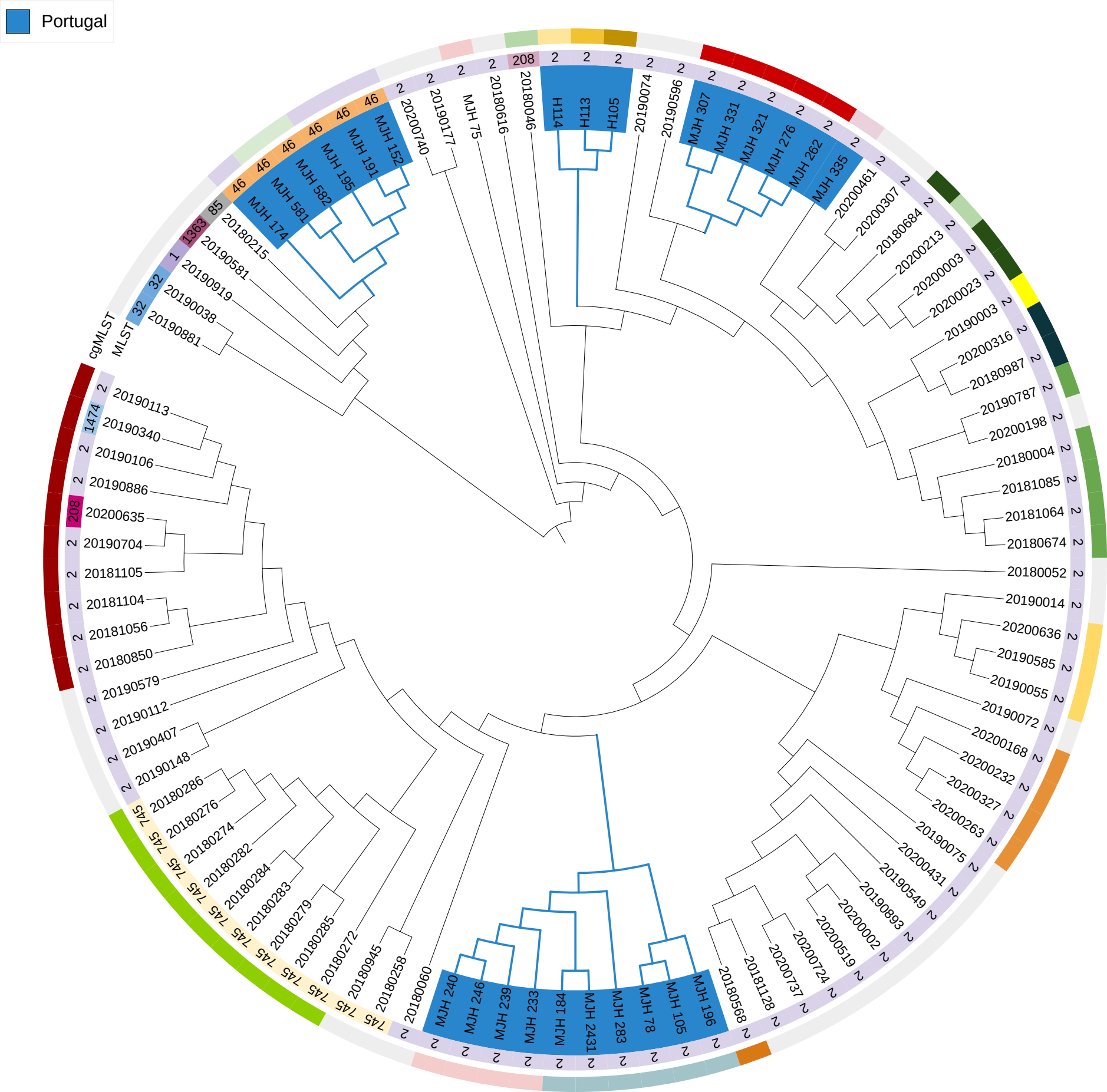

Supplement: Supplementary file 3 — Supplementary file2 Figure S2. Dendrogram generated with A. baumannii isolates from Andalucía and Portugal using the cgMLST scheme of Ridom SeqSphere+. (PDF 35 KB) [file 10096_2025_5047_MOESM2_ESM.pdf]
