# Supplementary material for: Regional distribution of carbapenemase-producing Acinetobacter baumannii isolates in southern Spain (Andalusia)
Source: Eur J Clin Microbiol Infect Dis. 2025 Feb 17;44(5):1069–76. doi: 10.1007/s10096-025-05047-2 (PMC12062160; doi:10.1007/s10096-025-05047-2)
Supplement: Supplementary file 4 — Supplementary file3 Table S1. Incidence density of carbapenem-resistant A. baumannii isolates in Andalusia (Spain) and number of submitted isolates to the Regional Reference laboratory PIRASOA during December 2017-2020. Legend of Table S1 *No. of inpatients with A. baumannii infection/colonisation x 1000/No. of total stays during the year, according to PIRASOA program reported data (htpp://pirasoa.iavante.es/course/view.php?id=3§ion=2). NR= not reported to the PIRASOA program; SD= standard deviation. (DOCX 25.2 KB) [file 10096_2025_5047_MOESM3_ESM.docx]

**Table S1.** Incidence density of carbapenem-resistant *A. baumannii* isolates in Andalusia (Spain) and number of submitted isolates to the Regional Reference laboratory PIRASOA during December 2017-2020.

**Legend of Table S1**

*No. of inpatients with *A. baumannii* infection/colonisation x 1000/No. of total stays during the year, according to PIRASOA program reported data (htpp://pirasoa.iavante.es/course/view.php?id=3&section=2).
NR= not reported to the PIRASOA program; SD= standard deviation.

| **Province  (No. Isolates)** | **Hospital** | **No. of submitted isolates** | **Annual mean of incidence density of inpatients with  carbapenemase-resistant *A. baumannii** (SD)** | | | |  |  |  |  |  |
| --- | --- | --- | --- | --- | --- | --- | --- | --- | --- | --- | --- |
|  |  |  | **2018** | **2019** | **2020** | **Mean** |  |  |  |  |  |
| Almería (n=120) | Hospital de Poniente (HP) | 1 | 0,00 (0,00) | 0,00 (0,00) | 0,01 (0,03) | 0,00 (0,01) |  |  |  |  |  |
|  | Hospital La Inmaculada (HHO) | 69 | **0,53 (0,49)** | **1,18 (0,19)** | **0,25 (0,06)** | **0,65 (0,48)** |  |  |  |  |  |
|  | Hospital Torrecárdenas (HT) | 50 | 0,00 (0,00) | **0,11 (0,04)** | 0,03 (0,06) | 0,05 (0,06) |  |  |  |  |  |
| Cádiz (n=17) | Hospital Puerta del Mar (HPM) | 1 | 0,00 (0,00) | 0,02 (0,02) | 0,05 (0,04) | 0,02 (0,03) |  |  |  |  |  |
|  | Hospital Punta de Europa (HPE) | 16 | **0,13 (0,19)** | 0,03 (0,05) | NR | 0,08 (0,07) |  |  |  |  |  |
| Córdoba (n=1) | Hospital Infanta Margarita (HIM) | 1 | 0,00 (0,00) | 0,00 (0,00) | NR | 0,00 (0,00) |  |  |  |  |  |
| Granada (n=86) | Hospital Campus de la Salud (HCS) | 3 | **0,18 (0,10)** | 0,08 (0,06) | 0,02 (0,02) | 0,09 (0,08) |  |  |  |  |  |
|  | Hospital Virgen de las Nieves (HVN) | 83 | 0,06 (0,05) | 0,06 (0,03) | 0,08 (0,05) | 0,07 (0,09) |  |  |  |  |  |
| Huelva (n=34) | Hospital Infanta Elena (HIE) | 34 | **0,51 (0,21)** | **0,12 (0,11)** | 0,08 (0,00) | **0,24 (0,24)** |  |  |  |  |  |
| Jaén (n=4) | Hospital de Jaén (HJ) | 1 | **0,13 (0,20)** | 0,01 (0,01) | NR | 0,07 (0,09) |  |  |  |  |  |
|  | Hospital Alto Guadalquivir (HAG) | 3 | 0,05 (0,10) | 0,05 (0,11) | 0,00 (0,00) | 0,03 (0,03) |  |  |  |  |  |
| Málaga (n=6) | Hospital Virgen de la Victoria (HVV) | 6 | 0,03 (0,04) | 0,00 (0,00) | 0,00 (0,00) | 0,01 (0,02) |  |  |  |  |  |
| Sevilla (n=68) | Hospital de Valme | 3 | 0,00 (0,00) | 0,01 (0,02) | 0,00 (0,00) | 0,00 (0,00) |  |  |  |  |  |
|  | Hospital Virgen Macarena (HVM) | 45 | 0,01 (0,02) | 0,04 (0,03) | 0,04 (0,06) | 0,03 (0,02) |  |  |  |  |  |
|  | Hospital San Lázaro (HSL) | 2 | NR | NR | NR | NR |  |  |  |  |  |
|  | Hospital San Juán de Dios Aljarafe (HSJDA) | 8 | 0,02 (0,04) | 0,00 (0,00) | 0,01 (0,01) | 0,01 (0,01) |  |  |  |  |  |
|  | Hospital San Juán de Dios Sevilla (HSJDS) | 10 | 0,02 (0,04) | 0,00 (0,00) | 0,01 (0,01) | 0,01 (0,01) |  |  |  |  |  |
|  | | | | | | |  |  |  |  |  |
|  |  |  |  |  |  |  |  |  |  |  |  |
