# Supplementary material for: Regional distribution of carbapenemase-producing Acinetobacter baumannii isolates in southern Spain (Andalusia)
Source: Eur J Clin Microbiol Infect Dis. 2025 Feb 17;44(5):1069–76. doi: 10.1007/s10096-025-05047-2 (PMC12062160; doi:10.1007/s10096-025-05047-2)
Supplement: Supplementary file 5 — Supplementary file4 Table S2. Assembly summary report for the 73 genomes of Acinetobacter spp., including 72 isolates of A. baumannii and one isolate of A. pittii. Legend of Table S2 *Isolate of A. pittii. (DOCX 25.0 KB) [file 10096_2025_5047_MOESM4_ESM.docx]

**Table S2.** Assembly summary report for the 73 genomes of *Acinetobacter* spp., including 72 isolates of *A. baumannii* and one isolate of *A. pittii*.

**Legend of Table S2**

*Isolate of *A. pittii*

| **Isolate ID** | **N75** | **N50** | **N25** | **Average** | **No. of contigs** | **Genome length** | **% of good targets*** |
| --- | --- | --- | --- | --- | --- | --- | --- |
| 20180004 | 87.127 | 174.666 | 234.279 | 14.876 | 268 | 3.986.812 | 99.6 |
| 20180046 | 93.204 | 174.388 | 234.296 | 66.405 | 59 | 3.917.921 | 98.7 |
| 20180052 | 67.677 | 161.738 | 232393 | 27.974 | 141 | 3.944.394 | 99.4 |
| 20180060 | 50.243 | 110.231 | 189.435 | 42.260 | 91 | 3.845.663 | 99.5 |
| 20180215 | 76.754 | 103.908 | 171.129 | 50.620 | 80 | 4.049.637 | 98.2 |
| 20180258 | 58.430 | 102.406 | 163.955 | 30.879 | 128 | 3.952.566 | 99.5 |
| 20180272 | 48.851 | 103.532 | 216.488 | 15.605 | 246 | 3.838.787 | 99.2 |
| 20180274 | 54.012 | 90.221 | 122.921 | 42.677 | 90 | 3.840.930 | 99.3 |
| 20180276 | 63.893 | 98.598 | 136.948 | 42.227 | 91 | 3.842.625 | 99.4 |
| 20180279 | 55.483 | 103.593 | 152.773 | 40.011 | 96 | 3.841.058 | 99.4 |
| 20180282 | 67.147 | 98.568 | 163.956 | 49.236 | 78 | 3.840.420 | 99.5 |
| 20180283 | 63.324 | 102.120 | 150.505 | 41.798 | 92 | 3.845.434 | 99.4 |
| 20180284 | 67.027 | 111.826 | 177.597 | 44.024 | 87 | 3.830.086 | 99.4 |
| 20180285 | 55.485 | 103.595 | 206.324 | 43.698 | 88 | 3.845.466 | 99.4 |
| 20180286 | 63.741 | 98.568 | 177.598 | 44.691 | 86 | 3.843.458 | 99.5 |
| 20180568 | 61.881 | 129.265 | 209.740 | 26.035 | 151 | 3.931.357 | 96.2 |
| 20180616 | 39.067 | 102.748 | 150.666 | 27.152 | 149 | 4.045.614 | 99.0 |
| 20180674 | 74.407 | 168.914 | 234.211 | 62.004 | 63 | 3.906.223 | 99.4 |
| 20180684 | 93.095 | 174.353 | 234.306 | 77.731 | 50 | 3.886.574 | 99.5 |
| 20180850 | 55.788 | 90.238 | 165.961 | 34.899 | 113 | 3.943.550 | 99.4 |
| 20180945 | 50.258 | 80.838 | 139.329 | 34.738 | 115 | 3.994.827 | 99.4 |
| 20180987 | 104.869 | 164.819 | 234.283 | 68.026 | 58 | 3.945.533 | 99.5 |
| 20181056 | 54.038 | 89.008 | 166.065 | 148.54 | 142 | 3.943.069 | 99.3 |
| 20181064 | 71.587 | 122.431 | 193.597 | 62.040 | 63 | 3.908.544 | 99.3 |
| 20181085 | 30.460 | 53.540 | 91.362 | 22.658 | 172 | 3.897.116 | 97.9 |
| 20181104 | 54.038 | 90.238 | 165.961 | 147.27 | 144 | 3.947.427 | 99.3 |
| 20181105 | 50.371 | 96.661 | 177.598 | 41.601 | 90 | 3.744.108 | 98.6 |
| 20181106* | 79.556 | 173.163 | 241.032 | 69.610 | 60 | 4.176.583 | 99.2 |
| 20181128 | 56.799 | 113.188 | 181.307 | 41.481 | 95 | 3.940.722 | 99.0 |
| 20190003 | 67.681 | 155.874 | 220.279 | 62.055 | 63 | 3.909.474 | 98.8 |
| 20190014 | 56.135 | 111.920 | 173.043 | 41.375 | 97 | 4.013.327 | 98.6 |
| 20190038 | 200.141 | 356.234 | 535.676 | 87.044 | 46 | 4.004.031 | 99.1 |
| 20190055 | 67.677 | 152.154 | 212.131 | 37.735 | 108 | 4.075.398 | 99.5 |
| 20190072 | 61.980 | 148.891 | 209.777 | 44.812 | 92 | 4.122.711 | 99.3 |
| 20190074 | 94.442 | 161.495 | 304.345 | 81.633 | 48 | 3.918.377 | 99.3 |
| 20190075 | 73.111 | 152.412 | 305.911 | 56.326 | 68 | 3.830.192 | 99.5 |
| 20190106 | 50.369 | 88.670 | 152.439 | 19.797 | 198 | 3.919.803 | 99.2 |
| 20190112 | 42.019 | 82.774 | 165.274 | 31.179 | 128 | 3.990.927 | 99.1 |
| 20190113 | 51.087 | 89.008 | 165.961 | 13.602 | 291 | 3.958.201 | 99.3 |
| 20190148 | 45.097 | 89.007 | 122.190 | 30.257 | 128 | 3.872.842 | 99.1 |
| 20190177 | 65.861 | 134.000 | 164.847 | 30.761 | 127 | 3.906.633 | 99.4 |
| 20190340 | 47.488 | 82.578 | 119.012 | 20.344 | 187 | 3.804.242 | 99.2 |
| 20190407 | 57.481 | 98.569 | 165.506 | 31.585 | 122 | 3.853.353 | 99.0 |
| 20190549 | 65.448 | 152.154 | 214.476 | 45.047 | 85 | 3.828.979 | 98.0 |
| 20190579 | 47.451 | 121.857 | 177.630 | 10.711 | 368 | 3.941.745 | 99.6 |
| 20190581 | 156.580 | 356.857 | 471.968 | 14.350 | 269 | 3.860.272 | 98.0 |
| 20190585 | 48.576 | 152.307 | 206.979 | 5.201 | 821 | 4.270.410 | 99.1 |
| 20190596 | 103.474 | 161.593 | 346.672 | 16.388 | 241 | 3.949.389 | 99.6 |
| 20190704 | 27.065 | 57.785 | 94.243 | 5.522 | 740 | 4.086.407 | 98.0 |
| 20190787 | 74.407 | 156.013 | 234.394 | 69.355 | 55 | 3.814.539 | 98.9 |
| 20190881 | 163.557 | 356.235 | 371.913 | 81.704 | 49 | 4.003.476 | 99.0 |
| 20190886 | 36.518 | 70.209 | 112.548 | 28.624 | 133 | 3.807.010 | 97.0 |
| 20190893 | 55.062 | 139.718 | 181.111 | 40.364 | 96 | 3.874.973 | 98.5 |
| 20190919 | 58.481 | 133.248 | 236.243 | 39.905 | 98 | 3.910.691 | 99.2 |
| 20200002 | 56.390 | 101.050 | 160.789 | 39.433 | 99 | 3.903.822 | 98.7 |
| 20200003 | 73.829 | 161.493 | 234.361 | 71.325 | 53 | 3.780.233 | 99.3 |
| 20200023 | 93.204 | 161.495 | 234.212 | 66.541 | 57 | 3.792.850 | 99.4 |
| 20200168 | 56.950 | 160.720 | 231.617 | 27.416 | 145 | 3.975.318 | 99.2 |
| 20200198 | 119.036 | 174.869 | 234.273 | 69.010 | 56 | 3.864.546 | 98.8 |
| 20200213 | 74.480 | 163.768 | 234.211 | 63.056 | 60 | 3.783.337 | 99.3 |
| 20200232 | 65.269 | 146.854 | 209.720 | 40.507 | 100 | 4.050.659 | 99.3 |
| 20200263 | 67.684 | 163.179 | 220.413 | 38.336 | 106 | 4.063.587 | 99.3 |
| 20200307 | 92.303 | 148.885 | 193.584 | 50.574 | 74 | 3.742.511 | 96.7 |
| 20200316 | 73.831 | 174.521 | 234.274 | 66.013 | 59 | 3.894.740 | 99.5 |
| 20200327 | 64.230 | 148.030 | 214.551 | 37.813 | 107 | 4.045.948 | 99.3 |
| 20200431 | 48.572 | 113.247 | 173.644 | 35.883 | 107 | 3.839.523 | 98.7 |
| 20200461 | 74.714 | 147.341 | 234.209 | 52.221 | 71 | 3.707.708 | 95.0 |
| 20200519 | 56.799 | 129.637 | 237.261 | 42.158 | 92 | 3.878.515 | 99.0 |
| 20200635 | 35.317 | 71.669 | 139.792 | 28.361 | 136 | 3.857.149 | 99.2 |
| 20200636 | 57.624 | 113.188 | 172.897 | 37.682 | 107 | 4.031.936 | 99.4 |
| 20200724 | 61.813 | 146.854 | 192.645 | 39.028 | 100 | 3.902.751 | 99.0 |
| 20200737 | 57.391 | 135.378 | 211.944 | 40.218 | 97 | 3.901.104 | 99.0 |
| 20200740 | 71.131 | 146.856 | 237.263 | 48.019 | 76 | 3.649.443 | 97.5 |

*Percentage of good targets using the Ridom SeqSphere+ cgMLST scheme.
